# Community effectiveness of indoor spraying as a dengue vector control method: A systematic review

**DOI:** 10.1371/journal.pntd.0005837

**Published:** 2017-08-31

**Authors:** Moody Samuel, Dorit Maoz, Pablo Manrique, Tara Ward, Silvia Runge-Ranzinger, Joao Toledo, Ross Boyce, Olaf Horstick

**Affiliations:** 1 Institute of Public Health, University of Heidelberg, Heidelberg, Germany; 2 Public Health Consultant, Muenchen, Germany; 3 Universidad Autónoma de Yucatán, Mérida, México; 4 Public Health Consultant, Accra, Ghana; 5 Public Health Consultant, Ludwigsburg, Germany; 6 Ministry of Health, Brasilia, Brazil; 7 Division of Infectious Diseases, University of North Carolina at Chapel Hill, Chapel Hill, United States of America; University of California, Davis, UNITED STATES

## Abstract

**Background:**

The prevention and control of dengue rely mainly on vector control methods, including indoor residual spraying (IRS) and indoor space spraying (ISS). This study aimed to systematically review the available evidence on community effectiveness of indoor spraying.

**Methods:**

A systematic review was conducted using seven databases (PubMed, EMBASE, LILACS, Web of Science, WHOLIS, Cochrane, and Google Scholar) and a manual search of the reference lists of the identified studies. Data from included studies were extracted, analysed and reported.

**Results:**

The review generated seven studies only, three IRS and four ISS (two/three controlled studies respectively). Two IRS studies measuring human transmission showed a decline. One IRS and all four ISS studies measuring adult mosquitoes showed a very good effect, up to 100%, but not sustained. Two IRS studies and one ISS measuring immature mosquitoes, showed mixed results.

**Conclusions:**

It is evident that IRS and also ISS are effective adulticidal interventions against *Aedes* mosquitoes. However, evidence to suggest effectiveness of IRS as a larvicidal intervention and to reduce human dengue cases is limited–and even more so for ISS. Overall, there is a paucity of studies available on these two interventions that may be promising for dengue vector control, particularly for IRS with its residual effect.

## Author summary

The effectiveness of indoor residual spraying (IRS) and indoor space spraying (ISS) as dengue vector control methods depends on many factors. This study aims to systematically review the evidence on the community effectiveness of indoor spraying of insecticides to reduce *Aedes* mosquito populations and thereby to control dengue transmission. A systematic literature review was performed in PubMed, EMBASE, LILACS, Web of Science, WHO library database (WHOLIS), Cochrane, and Google Scholar, including a manual search of the reference lists of the identified studies since its inceptions until 15.02.2017. A total of 39 articles were retrieved for full assessment. Seven studies were included and analysed after final application of inclusion and exclusion criteria: two IRS studies with control, one without, three ISS studies and one, respectively. One IRS study and four ISS studies showed good evidence of effectiveness on adult *Aedes* mosquitoes. Evidence of effectiveness of IRS as a larvicidal intervention exists but is still inadequate, and is weak for ISS. Evidence of effectiveness of IRS on human dengue cases as a single intervention exists, but was limited and not available for ISS. It is recommended to scale up the research regarding the community effectiveness of IRS and ISS, including measuring dengue transmission, particularly, for IRS with its residual effect. It is also suggested to study in depth the factors that could affect the community effectiveness of IRS and ISS on *Aedes* populations and on human dengue cases.

## Introduction

Dengue is the most prevalent arthropod-borne viral disease, infecting 300 to 500 million individuals each year. Approximately 100 million infections are symptomatic, which can range from mild to severe disease [1,2,3]. An estimated 500 000 people suffer from the severe forms, nearly 90% of whom are children, with a resulting 22 000 dengue-related deaths annually [4]. Global climate change, urbanisation, travel, poor sanitation, and inadequate public health services, all have the potential to increase the intensity of dengue transmission [5,6].

The four serotypes of the dengue virus (DENV 1–4) are transmitted principally by female *Aedes aegypti* and to a lesser extent by *Aedes albopictus* mosquitos [7]. *Aedes* species are anthropophilic, feed in the dark, the early morning and twilight hours and show an indoor-resting behaviour preferentially in secluded stationary locations e.g. under furniture, lower walls, under sinks, in curtain folds, or in wardrobes [2,8,9]. Dzul-Manzanilla [10] determined that *Aedes aegypti* rested mostly below 1.5 meters of height, and mostly in bedrooms (44%), living rooms (25%) and bathrooms (20%).

At present, there is no effective vaccine available, for public health use, to prevent or treat dengue infections, efficacy of the existing vaccine is variable and not high [11,12]. Therefore, vector control is the primary method of dengue prevention and control. Since the turn of the 19th century, chemical insecticides applied to the environment in a variety of methods have served as one of the mainstays of dengue vector control programmes, basically outdoors against immature and indoors-outdoors against adult vectors.

Indoor application of insecticides (IAI) includes indoor space spraying (ISS) or indoor residual spraying (IRS). Both target the endophilic adult *Aedes* mosquitoes that bite and rest indoors [10,13]. IRS entails the coating of walls and surfaces of the entire house with a residual insecticide [14]. ISS is done to treat indoor spaces to control flying insects with less residual effect. IRS can potentially target *Aedes aegypti* as it was used for the first time in Malaysia in 1952 [15]. IRS, however, is not generally recommended for dengue vector control, as it is thought that adult *Aedes aegypti* often rest on non-sprayable surfaces in houses [16]. Despite this, reductions in *Aedes aegypti* populations have been observed in areas where IRS is utilised for malaria control. A recent meta-analysis [17] concluded that there is a need of more empirical evidence supporting the potential utility of IRS for dengue prevention, since it was based on only two studies. For a meta-analysis comparability of studies precludes inclusion of many articles, thus providing a justification with an update and further inclusion and analysis of studies using IRS/ISS with a further systematic review.

This study systematically reviews the available evidence on community effectiveness of IRS and ISS for reducing *Aedes* populations and thereby for controlling dengue transmission.

## Methodology

This review follows the guidelines set forth in the PRISMA criteria for the reporting of systematic reviews and meta-analyses [18]. The literature search was conducted in parallel by two data extractors until 15.02.2017, with an update until 28.02.17.

A wide range of search terms was used in combinations to identify all relevant studies. The search terms included (a) disease specific terms: Dengue, Dengue hemorrhagic fever, Dengue haemorrhagic fever, Dengue shock syndrome, DHF, and DF, (b) vector specific terms: *Aedes*, *Aedes aegypti*, *Aedes albopictus*, *Ae*. *aeygpti*, and *Ae*. *albopictus*, and (c) intervention specific terms: Indoor space spray, ISS, Indoor residual spray, IRS, Residual house spray, and Intra domiciliary residual spray.

For the purposes of this review, IRS was defined as the application of chemical insecticides on walls and other surfaces with the aim to control *Aedes* mosquitoes inside houses, using substances which remain effective for 1 month or more. ISS was defined as any indoor spray using ultra-low volume spray (ULV), low-volume spray (LV), thermal fogging and other devices such as insecticide fumigant canisters. This review is limited to public health application of IRS/ISS, not commercial (household) use. Community effectiveness studies were defined as those studies conducted to evaluate the impact of IRS/ISS under normal field conditions, while efficacy studies were defined as those studies conducted under laboratory conditions.

The above search strategy was applied to the following databases: PubMed, EMBASE, LILACS, Web of Science, WHO library database (WHOLIS), Cochrane, and Google Scholar.

Eligible studies met the following inclusion criteria: 1) peer-reviewed publications presenting original data evaluating the community effectiveness of IRS/ISS 2) studies with control group(s) during intervention, studies with pre- and post-intervention assessments, and cross-sectional studies, 3) no language restrictions were applied, and 4) the target was vector and human populations. The exclusion criteria were limited to the following: 1) abstracts, conference posters, short communications, and letters to the editor, 2) studies with not enough information on community effectiveness of IRS/ISS, 3) efficacy studies and 4) surveillance data or reviews.

All identified studies were screened by title and abstract. Relevant studies were sent to EndNote X7 reference manager software. The numbers of relevant, irrelevant, and duplicated articles were identified and recorded for each database.

Full texts of selected studies were retrieved either through online databases or through Heidelberg University libraries. All reference lists of retrieved studies were screened for additional relevant studies. The full eligibility criteria were applied to all retrieved articles to identify the final list of included studies. The systematic literature search and the review followed the assessment of multiple system reviews, AMSTAR, for assuring the methodological quality [19].

By using a pre-designed extraction sheet, the following data were extracted from each included study: author name, year of publication, source database, study title, geographical location, objective(s) of the study, study design, relevant outcomes, main results, and key conclusions of the authors (Table 1).

**Table 1:** Evidence table.

**Indoor residual spraying**			
**Author, year of publication, study title**	**Objectives, study design, study setting**	**Sample size, outcome measures**	**Results**
**Controlled studies**			
**Parades-Esquivel (2015)** The impact of indoor residual spraying of deltamethrin on dengue vector populations in the Peruvian Amazon	To assess the impact of deltamethrin IRS on dengue vectors Intervention control trial Loreto, Peru	Intervention 36 houses: 12 constructed with painted wood, 12 with unpainted wood,12 with unpainted brick. Control Three houses (one per type of material) BI, CI, HI Adult indices	IRS reduced all immature indices in the first week after deltamethrin IRS application Adult index fell from 18.5 to 3.1, four weeks’ after intervention (p < 0.05)
**Vazquez-Prokopec (2010)** Quantifying the spatial dimension of dengue virus epidemic spread within a tropical urban environment	To assess the impact of IRS (Lambda-cyhalothrin) and spatial correlation in the odds of dengue infection Cross-correlation time series analysis, comparing to control (sprayed to non-sprayed houses) Cairns, North Queensland, Australia	383 DENV-2 confirmed cases and 1,163 IRS applications: 97 sprayed houses 151 non-sprayed houses Age adjusted dengue incidence Odds of secondary dengue infections	If IRS covered more than 60% of neighbouring premises: odds of secondary dengue infection at premises with confirmed dengue cases was significantly higher at unsprayed premises than at sprayed premises (OR = 2.8; 95% CI = 1.1–6.9; P = 0.03)
**Before and after studies**			
**Lien 1994** Dengue vector surveillance and control in Taiwan	To assess the effectiveness of IRS with alphacypermethrin as an emergency control measure Pre-and-post intervention Southern Taiwan	36977 sprayed houses 1991 14112 sprayed houses 1992 BI Larval density Number of confirmed and reported cases	BI from above 35 to under 5 Cases from above 3000 to under 1000
**Indoor space spraying**			
**Author, year of publication, study title**	**Objectives, study design, study setting**	**Sample size, outcome measures**	**Results**
**Controlled studies**			
**Mani (2005)** Efficacy of thermal fog application of deltacide, a synergized mixture of pyrethroids, against *Aedes aegypti*, the vector of dengue	To assess the effect of indoor and peridomestic spraying of deltacide on *Aedes* mosquitoes Intervention control trial Chennai, Tamil Nadu, India	3 residential colonies with 216–260 houses each 1 for peridomestic fogging 1 for indoor fogging 1 for control KD rates Adult mosquito densities % breeding sites BI	Adult mortality percentage reduction 100% post indoor fogging, 77.8% day 5, 6.25 day 7 BI 50 at baseline, post 7 days 29.6, post 14 days 37.5
**Perich (2003)** Evaluation of the efficacy of lambda-cyhalothrin applied by three spray application methods for emergency control of *Aedes aegypti* in Costa Rica	To assess the effect of lambda-cyhalothrin applied as ULV, LV and thermal fog spray against *Ae*. *aegypti* at front doors and inside rooms Intervention control trial Puntarenas, Costa Rica	Intervention 12 residential blocks 72 sprayed houses Control 2 residential blocks 12 untreated houses % adult mosquito mortality Adult density	Adult density dropped to 0 after spraying for thermal fog and ULV, increasing after day 7 and continued to increase until 7 weeks post spraying LV showed no significant difference to control
**Perich (2001)** Evaluation of the efficacy of lambda-cyhalothrin applied as ultra-low volume and thermal fog for emergency control of *Aedes aegypti* in Honduras	To assess the effect of lambda-cyhalothrin against *Ae*. *aegypti* when applied as ULV and thermal fog spray at front doors and inside rooms Intervention control trial El Progreso, Honduras	Intervention 4 residential blocks 24 treated houses Control 1 residential block 6 untreated houses Mean % mortality of adult mosquitoes Adult mosquito density	Adult density dropped to 0 for both treatments, increasing after day 7 and continued to increase until 7 weeks post spraying
**Before and after studies**			
**Koenraadt (2007)** Spatial and temporal patterns in the recovery of *Aedes aegypti* (Diptera: Culicidae) populations after insecticide treatment	To assess the effectiveness of insecticide applications in the field and to study different strategies of spraying against *Aedes* in both space and time (pyrethrin mixture, ULV) Pre-and-post intervention study Kamphaeng Phet province, Thailand	Four houses in two areas Adult mortality Adult mosquito density Parity rates Spatial and temporal relationship	Indoor spray reduced the number of adult mosquitoes to around 10%, however gradually recovering after day 2
**Further relevant studies published after initial searches**			
**Vazquez-Prokopec (2017)** Combining contact tracing with targeted indoor residual spraying significantly reduces dengue transmission.	To assess the effectiveness of IRS using space-time statistical data modelling with existing data Cairns, Australia	Data from 2008 and 2009 Probability of future DENV transmission	Data from 2008 and 2009 confirm that targeted IRS in potential exposure locations reduced the probability of future DENV transmission by 86 to 96%, compared to unsprayed premises

To assess for quality, included studies were categorised into studies with and without a control arm. They were further classified by study design and number of interventions. Outcome measures were extracted, classified and summarised across studies. Different measures were used to record the frequency of observations and the way of presentation varied according to the type of the presented data. Different insecticides and methods of application, together with varying statistical methods and outcome measures across the studies precluded any attempt at meta-analysis. Articles included through an update of the initial searches, until 28.02.17, are presented in the discussion section.

## Results

### Data search

**Fig 1:**
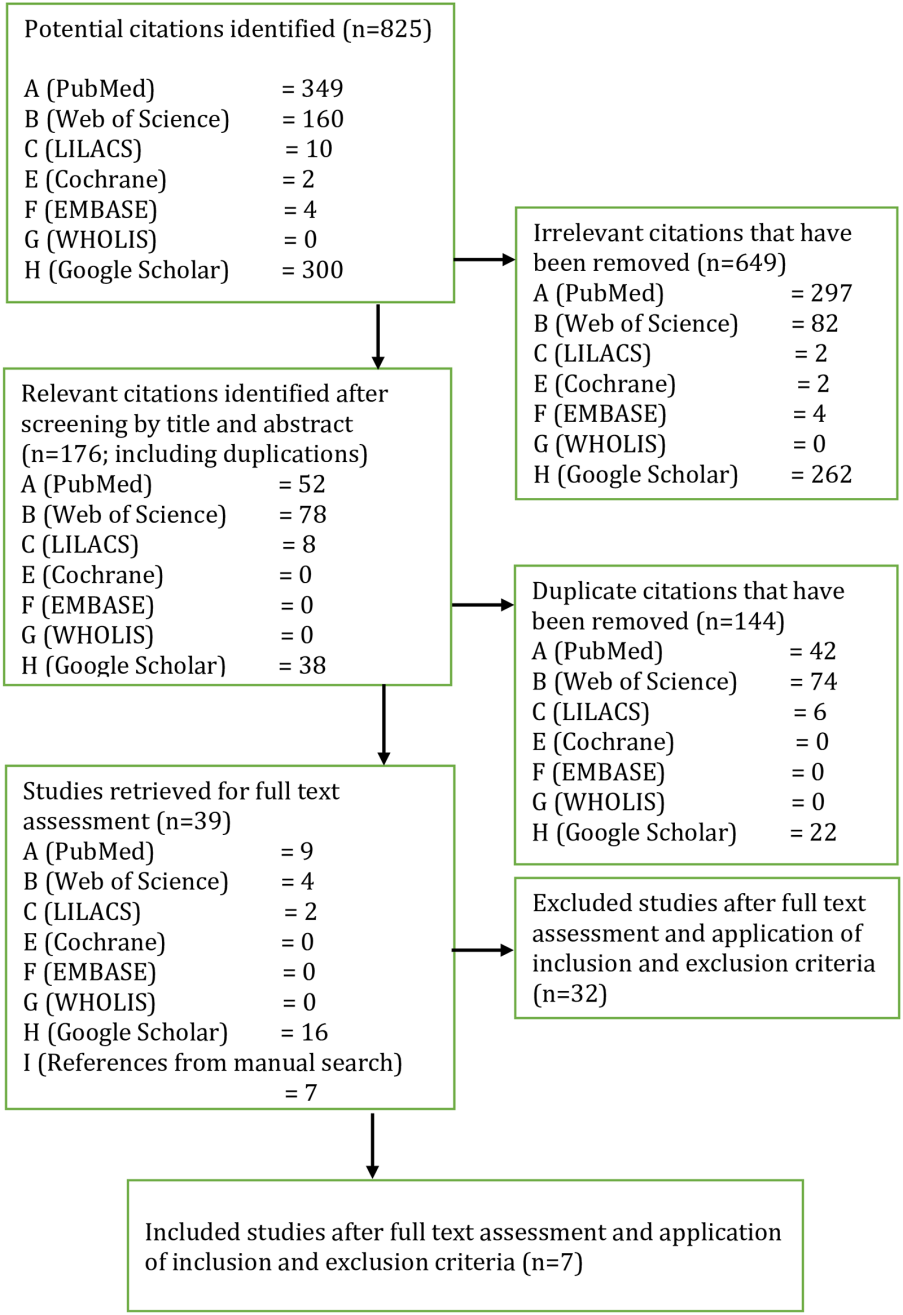
Flowchart of selection process.

A comprehensive literature search of the seven databases identified 825 potentially relevant citations. After screening for title and abstract, 144 duplicates and 649 irrelevant articles were excluded. The reference lists of the 32 remaining articles added seven more studies. The 39 studies were retrieved for full text assessment. Upon meeting eligibility criteria, seven studies were included and 32 studies were excluded, most of the latter were efficacy studies only (Fig 1). Summaries of included studies were arranged chronologically in an evidence table (Table 1).

### General characteristics

Seven studies met the pre-specified eligibility criteria (1) Three IRS studies: Parades-Esquivel 2015 [20], Vazquez-Prokopec 2010/1 [21], Lien 1994 [22]; 2) Four ISS studies: Mani 2005 [23], Perich 2003 [24], Perich 2001 [25] and Koenraadt 2007 26]). Most dengue risk areas were represented except Africa, with three studies from Asia [22,23,26], one study from Australia [21] and three from Latin America and the Caribbean [20,24,25]. All articles were reported in English. The time period of publication ranged from 1994 to 2015.

The seven studies were broadly classified into five controlled studies, two for IRS and three for ISS, and two non-controlled studies (one each IRS/ISS) (Table 1). Controlled studies were subsequently classified into four intervention control studies all testing IRS [20] and ISS [23,24,25]with multiple study arms. One cross-sectional time series compared data from sprayed and non-sprayed areas [21]. For the two non-controlled studies [22,26], Lien [22] had one study arm only, Koenraadt [26] had multiple study arms.

Reporting on sample size varied across included studies either for the diversity of methods or for the unavailability of data in some studies. For controlled studies, the smallest sample size for intervention was 36 houses [20] and the biggest three residential colonies with 216–260 houses each. The non-controlled studies covered 36977 houses [22] and four houses in two areas [26].

### Characteristics of study settings

All included studies reported on geographical locations. Parades-Esquivel [20], Vazquez-Prokopec [21], Mani [23] reported on meteorological conditions. All studies reported on the season/time period of the study, in relation to dry and rainy seasons. All studies discussed factors that might influence dengue transmission, and mosquito abundance, such as ecology and housing structures. The latter is described in detail by Parades-Esquivel [20], Vazquez-Prokopec [21] and Perich [24,25]. Parades-Esquivel [20] and Mani [23] present target populations and their socio-economic background. Pre-intervention dengue estimates were reported by Vazquez-Prokopec [21] and Lien [22]. Vazquez-Prokopec [21] reported on previous dengue outbreaks.

### Characteristics of the intervention

Method(s) of intervention: All studies used IRS as a single intervention. Although Vazquez-Prokopec [21] compared data from areas sprayed and not sprayed with IRS, in addition to the ongoing local control programme, including control of breeding places. Mani [23] compared ISS and peridomestic spraying, Perich [24] compared ISS with ULV, LV and thermal fogging and Perich [25] compared ISS with ULV and thermal fogging. Koenraadt [26] compared ISS and peridomestic spraying, with different insecticide concentrations.

Forms of application and formulations: Forms of application varied considerably, but including either ultra-low volume spray (ULV), thermal fog spray, or low-volume spray (LV). Formulations varied as well, including deltamethrin [20], lambda-cyhalothrin [21,24,25], alphacypermethrin [22], pyrethrin [26] and deltacide, a mixture of Deltamethrin 0.5%, S-Bioallethrin 0.75% and Piperonyl Butoxide 10% [23].

Duration of residual effect: Paredes-Esquivel [20] estimates a good residual effect of IRS up to 16 weeks, for ISS Perich [24,25] demonstrated three weeks and four weeks’ residual effect, respectively. Mani [23] and Koenraadt [26] showed a residual effect of one week.

### Control groups

Five of seven studies incorporated a control group into the study. They were assigned in different ways according to the methods used in each study. 1) IRS studies: Parades-Esquivel [20] used three single houses with similar structures to the 3 clusters of intervention houses (12 each). Vazquez-Prokopec [21] compared 97 sprayed houses to 151 non-sprayed houses, as the data were retrospectively available. 2) For ISS studies: Mani [23] used one cluster of houses (216–260) of three clusters for control. Perich [24] used two residential blocks with 12 untreated houses as control, Perich [25] used one residential bock with 6 untreated houses.

### Outcome measures

A variety of entomological and disease specific outcome measures were used to assess the impact of IRS: 1) Measures for adult *Aedes*: Adult mosquito mortality and knock down (KD) rates[20,23,24,25]; Adult mosquito density [20, 23,24,25,26] and spatial and temporal patterns [26]; 2) Measures for immature *Aedes*: Breteau Index (BI) [20,22,23]; House Index (HI) [20,22]; Percentages of breeding site [23]; Number of parous females [26]; 3) Disease specific measures: Age adjusted dengue incidence [21]; Odds of secondary dengue infection [21]; reported number of cases [22].

### Impact of indoor spraying of insecticides

The effect of indoor spraying of insecticides on adult mosquitoes is strong immediately after application in all studies measuring these parameters. For IRS studies, in Peru [20] the Adult Index fell from 18.5 to 3.1 four weeks’ after intervention (p < 0.05). For ISS studies, adult mortality percentage reduction was 100% post indoor spraying, 77.8% on day 5, 6.25 on day 7[23]. Similarly, adult density dropped to 0 after spraying with thermal fog and ULV, increasing after day 7 and continued to increase until 7 weeks post spraying, with similar results in Costa Rica [24] and Honduras [25]. In an uncontrolled setting in Thailand [26], indoor spraying reduced the number of adult mosquitoes to around 10%, however gradually recovering after day 2. The latter study measured also that there was a relationship between mosquito density and distance to the centre of application with an area of protection extending to 85 m. Parity rates also dropped after spraying.

The effect on immature mosquitoes is less strong on all studies measuring larval indices. For IRS studies, deltamethrin in Peru reduced all immature indices in the first week and sustained throughout the period of studies [20]. Also, there was a noted reduction of BI from 35 to 5 in Taiwan [22]. However, for ISS, in India, with a BI of 50 at baseline, this reduced to 29.6 post 7 days, and recovered post 14 days to 37.5.

For human dengue infection parameters, there are only two IRS studies. Odds of dengue infection shown by Vazquez-Prokopec [21], in Australia, were significantly higher at unsprayed than at sprayed premises (OR = 2.8; 95%CI = 1.1–6.9; p = 0.03). When 60% of the premises were sprayed around the index case house the odds reduced significantly to zero. Also the number of dengue cases was strongly and positively correlated to the number of IRS applications (r >0.6). Also, in Taiwan [22], the number of cases reported over time, dropped with IRS applications from above 3000 to 1000 (no control).

## Discussion

The evidence presented here suggests that IRS and ISS can be an effective dengue control intervention. The majority of included studies demonstrated a significant post-intervention reduction in adult and some effect on immature *Aedes* populations. Notably, of the studies that measured dengue incidence, both showed decreases in new dengue cases after the application of IRS. These findings support the use of IRS as a component of integrated vector management (IVM) [27], and perhaps ISS as well.

While the differing methodologies and interventions precluded meta-analysis, the included studies consistently show effective killing of adult *Aedes* mosquitos almost immediately after application of IRS and ISS. Estimates of the duration of effect are limited by the relative short time-frames studied, but multiple studies reported residual efficacy up to two months post-intervention. The impact of IRS on the incidence of dengue may be of even longer duration. The impact of ISS on dengue transmission was not measured. Further confirmations of the effect of IRS and ISS arise by two further studies [28,29]–the studies focused however on other elements and were excluded in the analysis. Ritchie [28] noticed an effect that started late but continued, using a combination of containers treated with S-methoprene or lambda-cyhalothrin and adult control with IRS using lambda-cyhalothrin, “human cases subsequently dropped from a high of seven cases per day in mid-March to only sporadic cases in late April, with the final reported onset of 7 May”. Stoddard [29] analysed surveillance data of dengue for explanatory models of transmission, ISS delivered in three cycles, using deltamethrin, cypermethrin, or alpha-cypermethrin, resulted in a good reduction of dengue transmission in trimester III. An update of the searches generated a further article, published shortly after the initial searches [30]. The authors conducted a study using space-time statistical data modelling from Cairns, Australia (data from 2008 and 2009). Targeted IRS “in potential exposure locations reduced the probability of future DENV transmission by 86 to 96%, compared to unsprayed premises”. This study strongly confirms the potential of IRS for reducing dengue transmission.

While there is evidence for indoor spraying in the control of dengue, there are a number of challenges with scaling up such interventions. Since, indoor spraying can require high levels of coverage, which requires widespread community acceptance and participation. Few studies included in the review reported qualitative estimates of community acceptance, although IRS is often popular as it has the ancillary benefit of killing many nuisance insects [1,4]. However, Chang [31] emphasised how communities are still reluctant to take appropriate dengue control measures. Furthermore, Gürtler [32] suggested integrating sustained social participation into IVM activities like source reduction, biological control, and environmental management, in order to overcome such a challenge and to ensure long-term sustainability of dengue prevention and control.

In addition, none of the included studies examined the associated costs of indoor spraying. In Australia however, where IRS is used for dengue control, a cost-analysis shows that the total costs of preparedness through surveillance are far lower than the ones needed to respond to the introduction of vector-borne pathogens [33]. Universal application and re-application is likely beyond the resources of many dengue-affected countries. Therefore, effective use of indoor spraying will require timely surveillance and response mechanisms. Combination of effective early warning systems with vector control measures could reduce densities of *Aedes* and subsequently dengue transmission [34]. Response systems could include mapping technologies like GIS [35]. Using space-temporal units besides such technologies is essential in delivering the resources and in measuring the coverage [36]. Analysis of one of the included studies showed similar evidence on how early detection of dengue outbreak helped to implement rapid and effective control actions, including early use of residual pesticides [22].

Experiments emphasised an association between type of insecticide used and its residual effect on *Aedes* and showed how the susceptibility of mosquitoes differs from one insecticide to another [37,38]. Perich [24,25] reported on another factor, which was the droplet size and linked it to post-spray residual effect. Sulaiman [39] pointed out how applying IRS on wooden surfaces is potentially controlling dengue. Another efficacy study in Malaysia linked house construction to the residual activity of IRS, since its wall bioassays indicated that both *Ae*. *aegypti* and *Ae*. *albopictus* were more susceptible to IRS on wooden surfaces than on brick surfaces [40]. Other challenges that are not well addressed in the included studies are optimal application and insecticide resistance, the latter is of a growing concern. Resistance particularly may affect severely the effectiveness of IRS/ISS. For residual treatment for example a study in Brazil showed a mortality of only 10% for *Aedes* in some communities for Deltamethrin [41].

A further challenge is the application of IRS, and where IRS is targeted. Whereas for Malaria and transmitting vectors IRS is defined as “the application of insecticide to the inside of dwellings, on walls and other surfaces that serve as a resting place for malaria-infected mosquitoes” and conditions for the use of IRS are set as “1) Majority of vectors (i.e., organisms that transmit malaria) must feed and rest indoors 2) Vectors are susceptible to the insecticide in use, 3) Houses have “sprayable” surfaces and 4) A high proportion of the houses in target areas are sprayed (more than 80 percent)” [42], such conditions are not as clear set for dengue vectors.

In addition to routine control measures, the use of indoor spraying as an emergency response is also feasible. Perich [24] pointed out how ISS successfully fulfils the criteria to be used as an emergency operation, which were: 1) providing an initial kill of adult *Aedes*, and 2) allowing a significant level of residual activity. Although residual activity with ISS may be mixed up with a time lag in recovery of mosquito populations. Evidence from that study and other efficacy studies in Malaysia and Taiwan plus ineffectiveness of outdoor spraying to control indoor *Aedes* populations make indoor spraying a true effective alternative for emergency suppression of *Aedes* mosquitoes [22,23,24,25,39,43]. This may also include the use of household (commercial) insecticides, another field that warrants analysis.

The key limitation of this systematic review is the very limited number of studies that typically researched community effectiveness of IRS and ISS. This study reports therefore on the different forms of application in relation to the outcomes. Also, potential publication and selection bias are most concerning. It is well documented that studies with positive outcomes are more often reported in literature than negative outcomes. The diversified and extensive search strategy along with no restrictions in languages should minimise the publication and selection bias.

The findings must also be interpreted with regard to the quality of the included studies: 1) Different methodologies, 2) Different study settings, 3) Limited use of statistical methods to assess for significance/control for confounding, 4) Relatively short study periods and 5) Lack of randomisation in most studies, influence the results.

However, the review is the most comprehensive to date and highlights the need for future work in this area. Concluding, evidence obtained from this systematic review showed that the use of IRS and ISS can produce significant reductions of *Aedes* populations (adult and immature forms). IRS can also produce significant reductions in human dengue cases, with very limited available evidence, but no data are available for ISS. However, evidence to suggest the effectiveness of IRS/ISS either on immature and adult stages of *Aedes* or on human dengue cases as a single intervention is limited.

The community effectiveness of IRS is affected, directly and indirectly, by many factors. Examples for these factors are disease epidemiology, virus dynamics, human movements, effective surveillance systems, community participation in vector control, the insecticides used, particularly considering insecticide resistance, environmental factors, and house construction. When these factors work in harmony with IRS/ISS applications, they would maximise its community effectiveness. Moreover, they could maximise the applicability of IRS/ISS, also being used as an emergency control measure during epidemics instead of being just applied as a routine control measure.

## Supporting information

S1 PRISMA Checklist (DOCX)
